# How do the blind ‘see’? The role of spontaneous brain activity in self-generated perception

**DOI:** 10.1093/brain/awaa384

**Published:** 2020-12-26

**Authors:** Avital Hahamy, Meytal Wilf, Boris Rosin, Marlene Behrmann, Rafael Malach

**Affiliations:** 1 The Wellcome Centre for Human Neuroimaging, UCL Queen Square Institute of Neurology, University College London, London WC1N 3AR, UK; 2 Department of Neurobiology, Weizmann Institute of Science, Rehovot, 7610001, Israel; 3 Department of Clinical Neuroscience, Lausanne University Hospital (CHUV), Switzerland; 4 Department of Ophthalmology, Hadassah-Hebrew University Medical Center, Jerusalem, 91120, Israel; 5 Department of Ophthalmology, University of Pittsburgh Medical Center (UPMC), Pittsburgh, PA 15213, USA; 6 Department of Psychology and Neuroscience Institute, Carnegie Mellon University, Pittsburgh, PA 15213, USA

**Keywords:** spontaneous activity, Charles Bonnet syndrome, functional MRI, spontaneous behaviour, visual hallucinations

## Abstract

Spontaneous activity of the human brain has been well documented, but little is known about the functional role of this ubiquitous neural phenomenon. It has previously been hypothesized that spontaneous brain activity underlies unprompted (internally generated) behaviour. We tested whether spontaneous brain activity might underlie internally-generated vision by studying the cortical visual system of five blind/visually-impaired individuals who experience vivid visual hallucinations (Charles Bonnet syndrome). Neural populations in the visual system of these individuals are deprived of external input, which may lead to their hyper-sensitization to spontaneous activity fluctuations. To test whether these spontaneous fluctuations can subserve visual hallucinations, the functional MRI brain activity of participants with Charles Bonnet syndrome obtained while they reported their hallucinations (spontaneous internally-generated vision) was compared to the: (i) brain activity evoked by veridical vision (externally-triggered vision) in sighted controls who were presented with a visual simulation of the hallucinatory streams; and (ii) brain activity of non-hallucinating blind controls during visual imagery (cued internally-generated vision). All conditions showed activity spanning large portions of the visual system. However, only the hallucination condition in the Charles Bonnet syndrome participants demonstrated unique temporal dynamics, characterized by a slow build-up of neural activity prior to the reported onset of hallucinations. This build-up was most pronounced in early visual cortex and then decayed along the visual hierarchy. These results suggest that, in the absence of external visual input, a build-up of spontaneous fluctuations in early visual cortex may activate the visual hierarchy, thereby triggering the experience of vision.

## Teaser

Some blind individuals experience visual hallucinations. Hahamy et al. show that these hallucinations are likely subserved by spontaneous brain activity in early visual cortex, which ignites the entire visual hierarchy, as in typical vision. Spontaneous brain activity can thus evoke unprompted perceptual behaviour.

## Introduction

During rest and independent of any external stimulation, the brain evinces activation in a spontaneous manner (‘resting state’ activity) (Arieli *et al.*, 1995; Nir *et al.*, 2006; Fox and Raichle, 2007; Raichle, 2009; Harmelech and Malach, 2013; Moutard *et al.*, 2015). It has previously been proposed that a slow build-up of spontaneous activity can initiate unprompted (spontaneous) behaviour (Schurger *et al.*, 2012; Moutard *et al.*, 2015). This phenomenon has been classically observed in the readiness potential during decisions to move (Kornhuber and Deecke, 1965; Libet *et al.*, 1983; Libet, 1985; Soon *et al.*, 2008; Fried *et al.*, 2011), but has also been observed during other cognitive tasks (Gelbard-Sagiv *et al.*, 2008, 2018; Norman *et al.*, 2019; Broday-Dvir and Malach, 2020). However, in these studies, the mere instruction to generate behaviour spontaneously prevents behaviour from being purely unprompted. Furthermore, since these studies involved task performance, brain activity was likely to be affected by the task demands, rather than being entirely spontaneous. 

Here, we addressed the question of whether spontaneous brain activity evokes unprompted cognitive behaviours, by examining participants with Charles Bonnet syndrome (CBS). This condition is characterized by complex visual hallucinations in individuals with a marked visual impairment, in the absence of a cognitive or mental disorder (de Morsier, 1936, 1967; Teunisse *et al.*, 1996). As individuals with CBS typically are unable to control the occurrence or the content of these hallucinations, hallucinations are genuinely unprompted perceptual behaviour. Furthermore, as neurons deprived of external inputs demonstrate enhanced spontaneous brain activity (Echlin *et al.*, 1952; Loeser and Ward, 1967; Segal and Furshpan, 1990), it has been theorized that spontaneous activity in the deprived visual cortex of individuals with CBS may subserve hallucinations (Cogan, 1973; Burke, 2002; Plummer *et al.*, 2007; Reichert *et al.*, 2013).

This hypothesis is particularly appealing in light of studies showing that, in sighted individuals, spontaneous (resting-state) activity can activate the entire visual system in aspects that are similar to those induced by naturalistic visual stimuli (Nir *et al.*, 2006; Gilaie-Dotan *et al.*, 2013; Wilf *et al.*, 2017; Strappini *et al.*, 2018). It is therefore possible that, in the absence of visual input, the existing neural mechanism that typically underlies veridical vision, and spans the entire visual cortex, may instead be activated by random spontaneous fluctuations. The triggering of the existing network-wide neural cascade by spontaneous activity might explain why the resulting hallucinations in CBS can be as vivid as they are in normal vision (Teunisse *et al.*, 1996; Menon, 2005).

Support for this hypothesis is, however, lacking. Previous studies of CBS have associated hallucinations with isolated visual regions (Ffytche *et al.*, 1998; Adachi *et al.*, 2000; Santhouse *et al.*, 2000; Kazui *et al.*, 2009; Painter *et al.*, 2018), for example, in the surround of the fusiform gyrus (Ffytche *et al.*, 1998), rather than with the entire visual cortex. Yet, given the interconnected nature of visual areas (Smith *et al.*, 2009), and their hierarchical functional organization (Grill-Spector and Malach, 2004), it seems unlikely that local activity, which is robust enough to evoke vision, would remain confined to a small region. The role of spontaneous brain activity in evoking hallucinations is also unclear, since, on the one hand, recent imaging findings ascribed hallucinations (Vacchiano *et al.*, 2019) or abnormal visual responses (Painter *et al.*, 2018) in individuals with CBS to external stimulation. On the other hand, the isolated activity in the fusiform gyrus, described by Ffytche *et al.* (1998), appeared to build up slowly over time prior to hallucination onset, which aligns with the hypothesis that slow spontaneous fluctuations underlie the CBS hallucinations. In summary, as evident from the above, neither the spatial nor dynamic neural mechanisms of CBS have been fully clarified.

**Figure 1:**
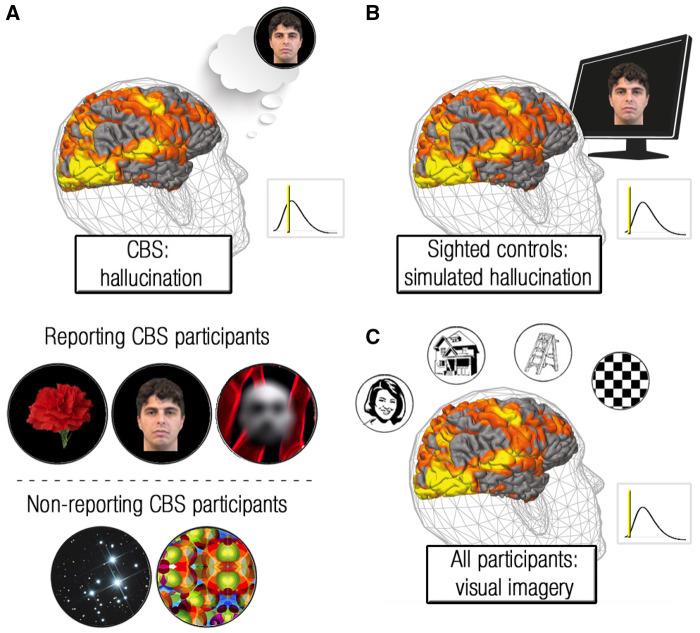
**Schematic of experimental design and participants.** (**A**) *Top*: Individuals with CBS were asked to verbally/manually report their visual hallucinations while in the MRI scanner. *Inset*: Schematic of blood oxygenation level-dependent (BOLD) signal in the visual system, vertical line represents the timing of hallucination report. The hypothesis for this condition is that activation in the visual system would ramp up prior to the perception of hallucinations. *Bottom*: Schematic of hallucination content of all CBS participants (Table 1). *Top row* depicts the hallucinatory content of the three CBS participants who were able to report their hallucinations; *bottom row* depicts the hallucinatory content of the two participants who were unable to report their hallucinations (see ‘Materials and methods’ section). (**B**) Verbal reports of hallucinations of the CBS participant were illustrated as movies, which were presented to sighted control participants. *Inset*: The experimental hypothesis of this condition is that activation in the visual system would ramp up only after visual stimuli are perceived. (**C**) All participants completed a visual imagery scan, in which they were asked to imagine faces, houses, objects and patterns. *Inset*: The hypothesis for this condition is that activation in the visual system would ramp up only after visual imagery has initiated. As depicted in this figure, visual hallucinations, like other visual experiences, were hypothesized to activate the entire visual hierarchy.

Here, we compared directly the neural correlates of hallucinations in individuals with CBS with the activation profiles evoked by veridical vision in sighted controls and by visual imagery in blind controls, across the entire visual cortex. As illustrated in Fig. 1, we predicted that (i) in the spatial (anatomic) domain, visual hallucinations would be associated with distributed activity across the visual system, similar to activations evoked by veridical vision in the sighted and by visual imagery in the blind (Cichy *et al.*, 2012; Dijkstra *et al.*, 2017, 2019); and (ii) in the temporal domain, unlike in other visual experiences, a build-up of activity in visual areas would precede the emergence of hallucinations. Such findings would suggest that temporal, rather than spatial, aspects of neural activity differentiate between unprompted visual hallucinations and other cued visual experiences.

## Materials and methods

### Participants

This study reports findings from five late-onset blind/visually-impaired individuals diagnosed with CBS (age 47 ± 8.9, two females, two left handed, one ambidextrous), 11 late-onset blind individuals not experiencing visual hallucinations (blind control group, age 40.54 ± 11, four females, two left handed, one ambidextrous) and 13 sighted individuals with normal or corrected-to-normal vision (sighted control group, age 43.85 ± 7.4, 10 females, two left handed). Experimental groups did not differ in age, gender or handedness (all *P*-values > 0.27, Fisher’s exact test). Participants had no history of psychiatric illness or cognitive impairments, and were not taking any psychoactive medications. Recruitment of participants was carried out with the assistance of a neuro-ophthalmologist, and through standard advertisements. All participants provided written informed consent before participating, in accordance with the Declaration of Helsinki, and were paid for their participation in the study. All procedures were approved by the Tel-Aviv Sourasky Medical Center, IRB ethics committee. Table 1 presents demographic, clinical and hallucination phenomenology information of the CBS participants (see also Supplementary material, ‘Hallucination phenomenology’ section).

**Table 1:** Phenomenology of hallucinations in participants with CBS

	CBS 1	CBS 2	CBS 3	CBS 4	CBS 5
Age	48	48	59	34	46
Gender	Female	Female	Male	Male	Male
Handedness	Ambidextrous	Right	Left	Left	Right
Visual acuity	No light perception	No light perception	Light perception	6/120	No light perception
Time since deterioration of vision, years	15	22	16	3	2.5
Diagnosis	Retinitis pigmentosa	Retinitis pigmentosa	Retinitis pigmentosa	Cone-rod dystrophy	Glaucoma
VVIQ score^a^	70	103	61	99	124
Hallucinatory content	Continuous stream of still images, including humans, animals, objects, houses, and patterns	Face of a man who is unfamiliar to the participant. The face can appear and disappear and can rotate or move across the visual field	Unfamiliar and distorted black and white faces, rotating objects, patterns and flashes of light	Very rapid flashes of light spanning the entire visual field	Continuous and rapidly changing shapes and colours that resemble kaleidoscope patterns
Frequency of hallucinations	Constant	Weekly	Every few months	Constant	Constant
Number of alternations in hallucinations per 8-min scan, mean ± SD	123.6 ± 50.5 (four verbal and four manual report scans)	13.7 ± 4.9 (two verbal and one manual report scans)	17.5 ± 12 (one verbal and one manual report scans)	Continuous	Continuous
Frequency of alternations in hallucinatory content	Seconds	Minutes	Seconds–minutes	Fractions of a second	Fractions of a second
Do hallucinations move with gaze?	No	Yes	Yes	Yes	Yes

a The possible range of the scores in the Vividness of Visual Imagery Questionnaire (VVIQ) across two administrations is 32–160, where low scores indicate higher imagery abilities.

### Experimental design

We aimed to study hallucinations (unprompted perceptual events) in CBS as a model for unprompted behaviours. To isolate the unprompted component of hallucinations, we compared hallucinations in CBS to cued veridical vision in sighted controls who were presented with visual simulations of these hallucinations. However, while the unprompted nature of visual hallucinations differentiates them from veridical vision, another difference is that hallucinations are internally generated while veridical vision is evoked by external stimulation. To control for this difference, we further compared hallucinations to cued visual imagery, as both of these conditions are internally generated, but only hallucinations are unprompted. Finally, to ensure that hallucination-related brain activations are not the product of mere verbal/manual report, we used a control condition consisting of verbal and manual tasks that were unrelated to the onsets of hallucinations.

The experimental procedures included the administration of questionnaires, and between one and three functional MRI sessions per participant. Session times ranged between 45 min and 120 min. In aggregate, these sessions included one resting-state scan (all groups), one to two imagery scans (all groups), an anatomical scan (all groups), a verbal-manual control condition (CBS participants/blind controls), and one visual localizer scan (sighted controls). We also conducted two to four hallucination scans reported verbally or using button-presses for each CBS participant, and for sighted controls, we conducted six simulated hallucination scans (as elaborated below). The order of scans was counterbalanced across participants with the exception that the first session always began with a resting-state run. Analyses of the visual localizer and resting state scans will not be reported here. Experimental software is detailed in the Supplementary material.

### Questionnaires

Questionnaires were used to collect demographic and clinical details. Additionally, the Vividness of Visual Imagery Questionnaire (VVIQ; Marks, 1973) was administered to assess how vividly participants imagined different scenes and situations. This questionnaire was administered twice, with participants' eyes being open/shut, and the scores across the two administrations were summed per participant.

### Hallucination report

During prescanning simulations, Participants CBS1–3 stated that they could report their hallucination as easily and promptly as they could identify visual stimuli before their vision deteriorated. Participants CBS4 and CBS5 said they were unable to report their hallucinations, and were therefore excluded from the report condition and subsequent analyses (but were included in all other conditions and analyses). The report condition consisted of several 8-min scans (number of scans depended on the availability/stamina of participants; Table 1), in which participants provided reports of their hallucinations either verbally, or via button presses. Because some CBS participants had some, albeit minimal, residual vision, participants were instructed to close their eyes during the entire scanning procedure. Participants were trained to speak without moving their heads, both outside and inside the scanner.

In-scanner verbal reports of hallucinations were recorded, and played back to the participants outside the scanner at the end of each session, asking them to give details of these hallucinatory events. Because the visual acuity of all CBS participants deteriorated at a relatively late stage of life, their description of the hallucinations was based on their prior visual experiences.

During the button-press runs, Participant CBS1 pressed a button using her index finger whenever an image appeared (note that hallucinatory images were constantly replaced by other images with no interval between them). Participants CBS2 and CBS3 pressed a button with the index finger whenever a face appeared and pressed a second button using the middle finger when the face disappeared.

### Simulated hallucinations

The temporal structure of the in-scanner verbal reports made by the CBS participants, along with the *post hoc* details regarding the hallucinations’ content, were used to create movies simulating these hallucinatory streams. Three such movies were created, one corresponding to each of the reporting CBS participants. The simulated hallucinations of Participant CBS1 consisted of images of humans, animals, body parts, objects, houses and patterns, presented in different sizes and positions on a grey background. The simulated hallucinations of Participant CBS2 consisted of a video recording of a male face, made small enough to move around the grey screen. The simulated hallucinations of Participant CBS3 consisted of various pictures and of video recordings of faces/patterns, all presented on a grey background. To account for the possible latency between the true onset of hallucinations and the actual reports made by CBS participants, all simulated stimuli were presented 1 s prior to their real temporal position, as reported by the CBS participants (Ben-Yakov and Henson, 2018).

Sighted control participants watched these simulated hallucination streams in an order that was counterbalanced across participants. Each simulated hallucination stream was watched twice, with instructions to report the hallucinatory content verbally or using button presses, as the CBS participants did. Participants were trained to speak without moving their heads. Two sighted control participants only completed the verbal-report runs, and additional four participants had one to three of their six scans excluded from further analyses because of excessive head motion. Nevertheless, all participants had at least one valid scan for each simulated hallucination stream.

### Visual imagery

All participants were asked to imagine faces, houses, objects and patterns. Before the scan, participants were given examples of items from each category. This 8-min run comprised 12-s blocks, each beginning with an auditory cue signalling a category name. These blocks ended with the auditory instruction ‘rest’, which was followed by an 8-s resting period. Block order was pseudo-randomized across participants. All participants closed their eyes during this experiment. CBS and all blind control participants completed two separate runs of this experiment (except for two blind controls, who completed only one run), and sighted controls completed one run. Data from one blind control were excluded from further analyses due to excessive head motion.

At the end of each run, participants assessed their success level in imagining each visual category on an increasing success scale of 1–5. These ratings were summed per participant.

### Verbal-manual control condition

To test whether verbal or manual reports alone evoke activity in the visual system, CBS and blind control participants performed a tone discrimination task. During this 8 min 6 s scan, participants heard a second-long tone of either 440 Hz or 460 Hz, interleaved with silent periods of 3–5 s. Stimulus order was pseudo-randomized across participants. Participants spoke/pressed a button when presented with the higher/lower frequency tone, respectively. Participants were trained to differentiate between the two tones before being scanned. Data from one blind control were excluded from further analyses due to excessive head motion.

See the Supplementary material for MRI data acquisition and preprocessing description.

### Statistical analysis

#### Questionnaires

Given the small sample size of participants, here and in all similar analyses, scores were compared between experimental groups using non-parametric permutation tests (Holmes *et al.*, 1996; Nichols and Holmes, 2002). Here, each test statistic was set to the difference between the group means. Under the null hypothesis of no group difference in imagery capabilities, participants’ group labels were shuffled to create two random groups of participants, and the difference between these groups’ means was calculated. This procedure was repeated for all possible permutations of participants between the two groups to construct the full null distribution, which was used to derive a two-tailed *P*-value for the true (unshuffled) test statistic.

#### Whole-brain analyses

To create task-based statistical parametric maps, we applied a voxel-based general linear model (GLM) as implemented in FSL’s FEAT, using a double-gamma haemodynamic response function convolved with the experimental model, as well as the resulting regressors' temporal derivatives. The six motion parameters and their derivatives, scrubbed volumes (Power *et al.*, 2012), and ventricle and white matter time courses for each participant (Fox *et al.*, 2009) were used as nuisance regressors. In addition, in the simulated hallucinations data, the first/last five repetition times (TRs) of each scan were included in the GLM model as nuisance variables, to remove the contribution of arousal-related effects. See the Supplementary material for a detailed description of all GLM designs.

As the sample size of the CBS group was small, whole-brain comparisons between the CBS group and any of the control groups were carried out using non-parametric randomization tests, as implemented in FSL’s randomize (Winkler *et al.*, 2014), including threshold free cluster enhancement correction for multiple comparisons. However, because of the inherent differences between the hallucination and the simulated hallucinations conditions (as it is impossible to simulate hallucinations with full precision), we refrained from directly contrasting activation strengths between these conditions, as any effects could be equally attributed to differences between hallucinations and veridical vision, or to differences in the visual stimuli.

Parametric activation maps were projected onto a template of a flattened cortical surface using the Connectome Workbench.

#### Quantifying the similarity between visual activations

Our hypothesis that hallucination-related activations would be similar to activations evoked by other visual experiences was tested in the posterior part of the brain (25 876 grey matter voxels corresponding to *y *<* *44 in MNI space; Gilaie-Dotan *et al.*, 2013). CBS hallucination activations (group beta values) were correlated with imagery/simulated-hallucinations beta values of each sighted control and with imagery/verbal-manual control condition beta values in each blind control. This calculation was performed twice for sighted controls in the simulated-hallucination condition: once modelled using the CBS report protocol, and once using a protocol locked to the sighted controls' own manual report. In both cases, for each sighted control participant, analysis was carried out using beta-value maps resulting from an FFX analysis of all three simulated-hallucinations data (corresponding to simulations of the three CBS participants’ hallucinations). Resulting correlation coefficients of the participants in each group and experimental condition were tested using a two-tailed one-sample Wilcoxon test.

Note that we refrained from statistically testing the posterior brain correlations of CBS participants across the different experimental conditions, because: (i) any significant similarities between the activations evoked by the imagery/control condition to those evoked by hallucinations may be confounded by the fact that CBS participants hallucinated during all conditions; and (ii) any absence of statistical significance could be due to the lack of statistical power in testing very small samples (specifically, the largest possible effect size in a sample of *n = *5 in a Wilcoxon test would correspond with a *P*-value of 0.03. Any smaller effect size would be non-significant under an alpha level of 0.05). Nevertheless, we assume that since CBS participants originate from the blind population, any effects found in blind controls during the imagery/verbal-manual control conditions should be representative of similar effects in CBS participants.

See the Supplementary material for a description of a bootstrap analysis testing whether the measured correlations were driven by noise.

#### Evaluation of temporal dynamics

To assess differences in the temporal dynamics of blood oxygenation level-dependent (BOLD) activity between the experimental groups, we extracted signals from an early/intermediate visual and a fusiform face area (FFA) regions of interest. Sensorimotor lip/hand regions of interest were also used as control regions for the verbal/manual report scans, respectively (see the Supplementary material for region of interest definitions and testing of activation in early/intermediate visual regions of interest).

Single participant’s signals were extracted from each region of interest, *z*-score normalized and subjected to an event related averaging analysis, within a time window of 3 TRs prior to stimulus onset to 7 TRs after stimulus onset. These signals were later averaged across participants of the same experimental group and for each experimental condition.

The BOLD signal typically rises shortly after stimulus presentation, but due to noise factors (e.g. slight asynchronies between scanner and experimental protocol, inconsistencies in participants’ attentiveness across trials, etc.) the event-related signals for individual participants may show slight random jitter (±1 TR) around the true event timings. Here, however, we had a clear prediction that the BOLD signal in visual regions across all CBS participants should consistently precede the reported onset of hallucinations, unlike in other experimental conditions or in the control groups. To test this prediction statistically, a canonical haemodynamic response function (HRF) was fitted to the event-related data of each individual participant (this was done automatically without the possibility of adjustment). This HRF was fitted to the data five times, each time with a different lag, ranging between 3 TRs prior to the modelled neural event (hallucination/imagery/vision) to 1 TR after the modelled neural event. The HRF lag that produced the best fit between the HRF and data was identified in each participant. These ‘optimal lags’ of the HRF in each region of interest were then compared between the CBS group and each of the sighted/blind control groups separately, using a permutation test (Supplementary material).

All sighted controls' simulated-hallucinations data (verbal and button-press scans) were analysed using a protocol locked to the hallucination report of CBS participants, and button-press scans were further analysed using a protocol locked to the individual button presses of each sighted control participant. The analysis of the sensorimotor lips/hand regions of interest made use of scans involving verbal/button-press reports, respectively.

#### Quantifying BOLD temporal dynamics across the visual hierarchy

To assess differences in the onset of the BOLD responses across regions of the visual system, all regions of interest of a probabilistic atlas (Wang *et al.*, 2015) were ranked based on their position in the visual hierarchy (Supplementary Table 1). The optimal HRF lag was calculated for each CBS participant and region of interest in the hallucination and imagery conditions, as explained earlier. Then, Spearman’s correlation was calculated between the rank of all regions of interest and the group-averaged optimal lags in these regions of interest. The resulting correlation coefficients were tested using a permutation test, under the null hypothesis of no correlation between ranks across the visual hierarchy and optimal lags. region of interest ranks across the visual hierarchy were therefore shuffled 10 000 times, and, each time, the correlation coefficient between the random ranks and optimal lags was computed. Two-tailed *P*-values were derived based on this null distribution.

### Data availability

Statistical data and experimental materials are available upon request.

## Results

Does spontaneous brain activity underlie deprivation-related visual hallucinations, and if so, by what mechanism? To answer these questions, five late-onset blind individuals with CBS, 11 late-onset blind controls who did not experience hallucinations, and 13 sighted control participants were recruited for an functional MRI study with three main conditions (Fig. 1). CBS participants verbally/manually reported their hallucinations while being scanned. A visual simulation composed of their hallucinatory streams was later presented to sighted controls. In addition, all participants completed a visual imagery scan.

### Behavioural imagery abilities

Imagery abilities, as assessed using the total score of the VVIQ questionnaire (Marks, 1973), did not differ between the CBS (91.4 ± 12.83) and either the blind (69.64 ± 8.34) or the sighted (78.15 ± 10.64) control groups (*P = *0.08 and 0.23, respectively, two-tailed permutation tests) (Table 1). Similarly, the in-scanner reports of the level to which participants succeeded in imagining visual categories during the experiment did not differ between the CBS (16.3 ± 1.6) and either the blind (17.45 ± 0.54) or the sighted (13.23 ± 1.06) control groups (*P = *0.2 and 0.08, respectively, two-tailed permutation tests).

### Hallucinations and veridical vision activate similar visual areas

**Figure 2:**
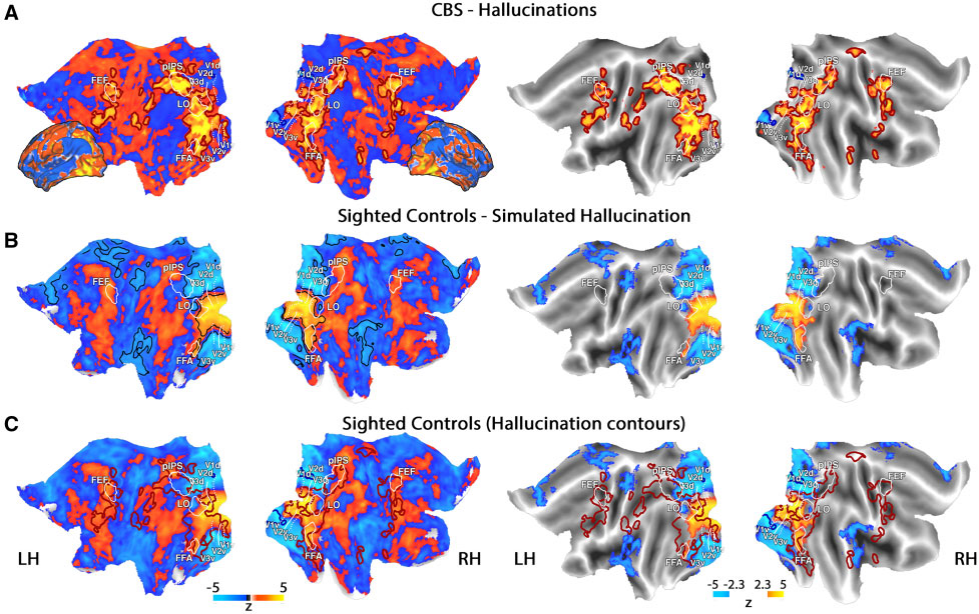
**Hallucination compared to normal vision.** *Left column*: Unthresholded maps; *right column*: The same maps with a statistical threshold, corrected for multiple comparisons. (**A**) CBS group maps in the hallucination condition (hallucination versus baseline), projected onto a representative flat cortical surface (the same map projected onto an inflated cortical surface is presented for reference). Wide red/blue contours depict significant areas of activation/deactivation, corrected for multiple comparisons. (**B**) Sighted control group maps in the simulated hallucination condition (simulated hallucinations versus baseline). Black contours depict significant areas of activation or deactivation, corrected for multiple comparisons. (**C**) The same group maps of the sighted controls in the simulated-hallucination condition, as presented in **B**, superimposed with the significant areas activated/deactivated during hallucinations in the CBS group (wide contours, as in **A**). White contours depict visual landmarks, based on a probabilistic atlas. FEF = frontal eye field; LH = left hemisphere; LO = lateral occipital complex; pIPS = posterior intraparietal sulcus; RH = right hemisphere. See Supplementary Figs 1 and 2.

We first aimed to compare brain activations evoked by hallucinations in the three CBS participants able to provide a reliable spatiotemporal description of their hallucinations (see ‘Materials and methods’ section and Table 1) to activations evoked by veridical vision in sighted controls. As depicted in Fig. 2A, a whole-brain analysis of hallucination-related activity in the CBS participants revealed significant activations across the entire visual hierarchy (see Supplementary Figs 1 and 2 for single CBS participants' maps). The simulated hallucinations in the sighted control group significantly activated the central visual field representations of early visual areas, as well as areas in the ventral stream, including the FFA. Peripheral visual field representations were significantly deactivated (Fig. 2B).

The apparent group difference in the spatial extent of visual activations between the CBS and sighted controls may have been the result of the difficulty in accurately simulating the internal visual experiences of the CBS participants. For example, because of technical limitations, some of the displays constructed for the simulated hallucinations were much smaller spatially than those reported by the CBS participants, and may thus have led to reduced activation in peripheral visual areas. Nevertheless, in line with our hypothesis that hallucinations and veridical vision would evoke a similar profile of brain activity (Fig. 1), superimposing the contours of significantly activated areas in the CBS group over the sighted controls' activation map, revealed that the two patterns of activity overlapped across many regions of the visual hierarchy (Fig. 2C). This observation was quantitatively supported by significant inter-group correlation of activity patterns across the entire posterior part of the brain (median of correlations = 0.2, df = 12, *P = *0.002, two-tailed Wilcoxon test). This profile of overlap was mostly unaltered when brain activations of sighted controls were not modelled based on the timings of reports made by CBS participants, but rather on the timing of reports made by the sighted controls themselves (median *=* 0.11, df = 12, *P = *0.006, two-tailed Wilcoxon test; Supplementary Figs 1 and 2. Note that modelling of the sighted controls' own reports was done on those scans in which participants reported manually, which comprised half of the data). Furthermore, these correlations were significantly greater than would have been expected by noise, as estimated using shuffled data (*P *<* *0.001/*P = *0.01 for CBS-locked/controls-locked protocols, respectively, two-tailed permutation tests).

It could be argued that the hallucination-related activations in the CBS group were not related to the self-generated visual precepts, but rather to the attentional and report-related aspects of the experiment. To test this possibility, CBS participants and blind controls completed a tone discrimination task in which they responded manually or verbally to specific tones. As presented in Supplementary Fig. 3A (also see Supplementary Figs 4 and 5 for maps of single CBS participants), blind controls showed weak activations in parts of the visual system during this task, which showed some resemblance to the spatial patterns of hallucination-related activations (median *=* 0.09, df = 9, *P = *0.05, two-tailed Wilcoxon test). These correlations were also greater than would have been expected by noise (*P = *0.01, two-tailed permutation test). A qualitative examination of Supplementary Fig. 3B and C revealed that these report-related activations were diminished in the CBS group map (here the ability to directly test for similarity to the spatial patterns of hallucination-related activations is compromised due to statistical/methodological reasons, see ‘Materials and methods’ section). A whole-brain between-group contrast revealed no significant differences between the CBS and blind control groups. Thus, some weak visual activations were evoked by auditory, verbal or motor processing in the blind control group, and perhaps in the CBS group as well. However, it seems unlikely that weak activations that appear in both groups would underlie the emergence of visual hallucinations, which occurs only in the CBS group.

### Hallucinations and visual imagery in the blind activate similar visual areas

**Figure 3:**
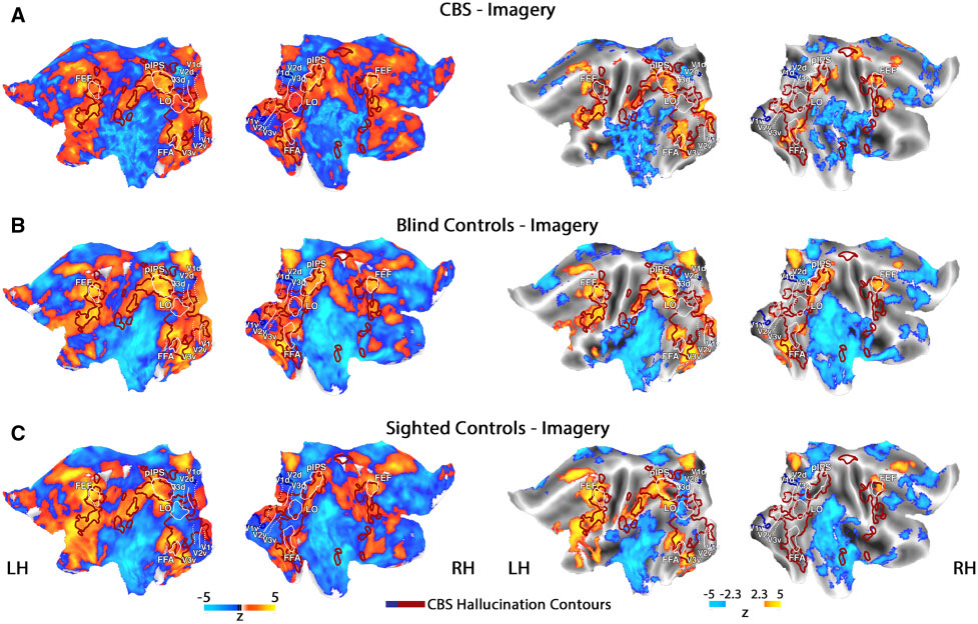
**Hallucination compared to visual imagery**. *Left column*: Unthresholded maps; *right column*: the same maps with a statistical threshold, corrected for multiple comparisons. Group maps of the CBS group (**A**), blind control group (**B**), and sighted control group (**C**) during the visual imagery condition (imagery versus baseline). These maps are overlaid with the contours of areas activated/deactivated during hallucinations in the CBS group (as presented in Fig. 2, wide contours). Note the spatial overlap between hallucination and imagery activations in both the CBS and blind control groups in the left panel. FEF = frontal eye field; LH = left hemisphere; LO = lateral occipital complex; pIPS = posterior intraparietal sulcus; RH = right hemisphere. See also Supplementary Figs 4 and 5.

The unprompted nature of visual hallucinations differentiates them from veridical vision. However, another difference is that hallucinations are internally generated while veridical vision is evoked by external stimulation. We next set out to compare hallucinations to cued visual imagery, as both are internally generated, but only hallucinations are unprompted. The maps of all experimental groups during the visual imagery condition are presented in Fig. 3 (also see single CBS participants' maps during visual imagery in Supplementary Figs 4 and 5). These maps display a network of visual and frontal areas, previously reported to be involved in visual imagery and visual perception in sighted controls (Behrmann, 2000; Ganis *et al.*, 2004; Yellin *et al.*, 2015; Dijkstra *et al.*, 2017). As depicted in Fig. 3, left, while the sighted control group tended to activate only higher-order visual areas and deactivate mid-level areas, both the CBS and blind control groups showed activations across the visual system, which reached significance in both lower- and higher-order visual areas. A direct comparison of whole-brain activations between the CBS and each of the control groups revealed no significant differences.f

As expected by the visual inspection of the sighted controls' group map (Fig. 3C), no evidence for similarity between imagery activations in the sighted controls and hallucination-related activations in the CBS group was found in the posterior part of the brain (median *=* 0.13, df = 12, *P = *0.09, two-tailed Wilcoxon test). However, a visual inspection of the left panel of Fig. 3 suggested that the contours of hallucination-activated areas in the CBS group largely matched the spatial activations seen during visual imagery in the CBS and blind control groups. This observation was confirmed quantitatively in the blind control group (median *=* 0.16, df = 9, *P = *0.002, two-tailed Wilcoxon test), and correlations were significantly greater than those expected by noise (*P *≤* *0.001, two-tailed permutation test). We note that the ability to test for similarities between the spatial activation patterns evoked by hallucinations and imagery in the CBS group is compromised because of statistical and methodological reasons (see ‘Materials and methods’ section). However, our results suggest that visual hallucinations in CBS and visual imagery in the blind (the population from which CBS emerges) tend to evoke similar spatial patterns of activations in the posterior brain.

### The temporal dynamics of hallucinations differ from those of veridical vision and visual imagery

We next hypothesized that although activations evoked by hallucinations show some spatial overlap with those evoked by veridical vision in the sighted and visual imagery in the blind, they may differ in their temporal dynamics. As schematically illustrated in Fig. 1, we expected that both the external perception and cued imagery of stimuli would bring about a rise in the BOLD response in visual areas. This would suggest that the optimal fit of an HRF to the data would be expected when there is a negligible lag (of no more than 1 TR) between the data and HRF. Contrary to this, we hypothesized that a rise in the visual BOLD response would precede hallucinations. This would require that the optimal fit of an HRF to the data would be at a negative lag, such that the BOLD signal begins rising prior to hallucination onset.

**Figure 4:**
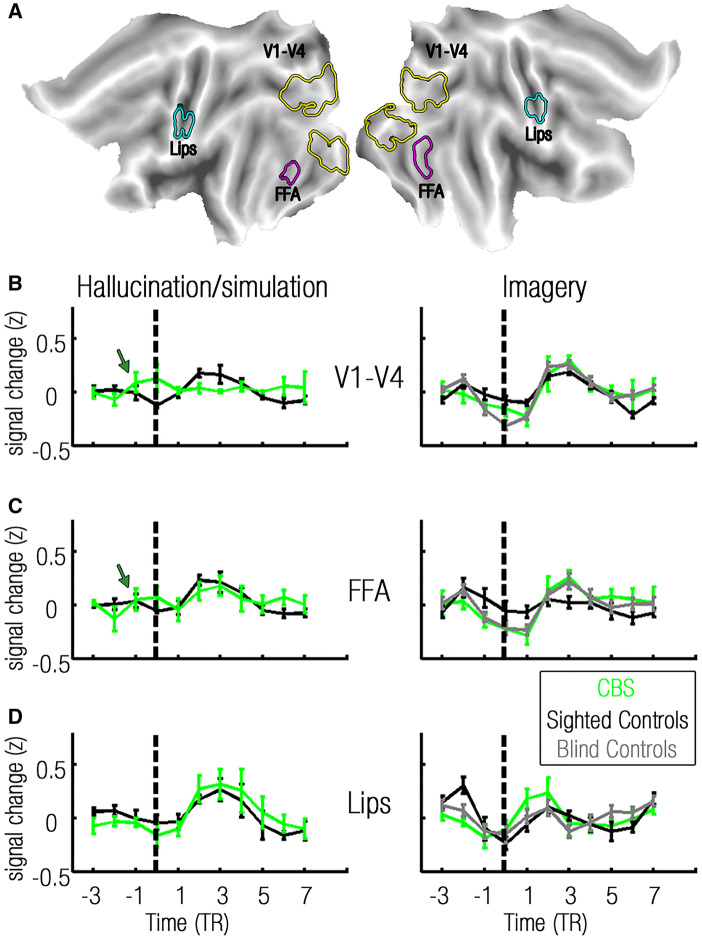
**Hallucination, vision and visual imagery have different temporal dynamics.** (**A**) Regions of interest V1–V4 (yellow contours), FFA (purple contours), lip areas (light blue contours). (**B**) Event-related averaging of group activations within each region of interest. Rows correspond with regions of interest (*top row*: V1–V4; *middle row*: FFA; and *bottom row*: lip region), and columns correspond with experimental conditions (hallucination in CBS participants and simulated hallucination in sighted controls in the *left column*, and visual imagery in the *right column*). Groups are represented by green, black and grey lines for CBS, sighted controls, and blind controls, respectively. Dashed lines depict hallucination onset (CBS), stimulus appearance in the visual condition (sighted controls) and onset of imagery instruction in the imagery condition. Note that visual (V1–V4, FFA) activations in the CBS group precede the onset of hallucinations, as marked with dark green arrows. This is unlike non-visual activations (lips region of interest) in the CBS group, simulated-hallucination activations in the sighted control group and imagery activations in all groups. See also Supplementary Figs 6–8.

To test whether the rise of the BOLD signal in CBS participants during the hallucination condition precedes that of controls during the simulated-hallucination condition (Fig. 1), we first examined the neural dynamics of a bilateral early/intermediate visual region of interest comprising areas V1–V4 (Fig. 4A), which showed a significant BOLD signal increase during hallucinations (*P = *0.04, one-tailed permutation test). As depicted in Fig. 4B, in the sighted control group, the appearance of stimuli in the simulated-hallucination condition was accompanied by a rise in the BOLD signal (mean optimal lag −0.26 TR). Importantly, and consistent with our hypothesis, the BOLD signal in the CBS group began building up 2.33 TRs on average before the reported onset of hallucinations in the early/intermediate visual region of interest. This difference in the temporal dynamics between the CBS and sighted control groups was statistically significant in this region of interest (*P = *0.002, one-tailed permutation test), and also in an additional early/intermediate visual region of interest composed only of voxels which were significantly activated by hallucinations (*P = *0.003, one-tailed permutation test). A similar effect was also observed when the modelled events in sighted controls were based on their own responses in the scans involving manual report (half the scans), rather than on the reports of the CBS participants across all scans (sighted controls optimal lag −0.97 TRs, *P = *0.056 one-tailed permutation test; Supplementary Fig. 6).

Importantly, the group difference in temporal dynamics was not observed during the cue-driven imagery scans. During imagery, the temporal dynamics of the CBS group (mean optimal lag 0.6 TRs) did not differ from those of sighted controls (mean optimal lag −0.75 TRs, *P = *0.09, two-tailed permutation test) or of blind controls (mean optimal lag: −0.3 TRs, *P = *0.07, two-tailed permutation test). A significant group (CBS, Sighted controls) × condition (Hallucination/simulated-hallucination, Imagery) interaction (sighted controls’ protocol locked to the CBS participants’ report: *P = *0.01; protocol locked to the sighted controls’ report: *P = *0.04, two-tailed permutation tests) indicated that the earlier build-up of activity in the CBS participants during hallucination (compared to veridical vision in the sighted controls) is not a general temporal characteristic of their visual system, but rather a unique manifestation of hallucination.

To examine activations in higher order visual regions, we chose the bilateral FFA (Fig. 4A), both because all reporting CBS participants hallucinated faces (Table 1), and because the fusiform gyrus has been previously reported to show a build-up of activity prior to the onset of hallucinations in CBS participants (Ffytche *et al.*, 1998). As depicted in Fig. 4C, a build-up of activity in the FFA did indeed precede hallucinations in the CBS group (mean optimal lag: −1 TR), replicating previous findings (Ffytche *et al.*, 1998). While this difference did not reach significance when the protocol of sighted controls was locked to the CBS participants’ report in both manual and verbal scans (sighted controls optimal lag: −0.3 TRs, *P = *0.32, one-tailed permutation test), significant results were found when using a protocol locked to the sighted controls' reports in the manual scans (sighted controls optimal lag: 0.3 TRs, *P = *0.04, one-tailed permutation test). This relatively weak effect is reflected in the bimodal shape of the averaged FFA signal, which captures the higher variability between the temporal dynamics of single CBS participants in this region compared to the dynamics in the early/intermediate visual region of interest (Supplementary Table 2). An opposite effect was observed in the visual imagery condition, as the mean optimal HRF lag of the CBS group (0.8 TRs) was significantly delayed compared to the mean optimal lag of the sighted control group (−1.1 TRs, *P = *0.01, two-tailed permutation test, though note this effect is likely due to the reduced FFA activation during imagery in the sighted controls). A similar effect was found when comparing the CBS to the blind control group (optimal lag: 0.3 TRs, *P = *0.04, two-tailed permutation test). This slightly delayed rise in signal in the CBS versus blind control participants may reflect slower initiation of imagery upon instruction in CBS participants, possibly due to interference of hallucinatory visual precepts. The observation of an earlier build-up of activity in CBS participants during hallucinations compared to imagery, and compared to sighted control participants was supported by a significant group (CBS, sighted controls) by condition (hallucination/simulated-hallucinations, imagery) interaction (sighted controls’ protocol locked to CBS report: *P = *0.02, protocol locked to the sighted controls’ report: *P *<* *0.001, two-tailed permutation tests).

Of significance, no build-up of activity in the CBS group was observed in areas engaged in the hallucination condition, but located outside the visual system proper. CBS participants showed a slightly delayed BOLD signal in the motor lips area (optimal lag: 0.33 TR) compared to sighted controls (optimal lag: −0.49 TR, *P = *0.03 two-tailed permutation test) in the scans involving verbal report of hallucinations. Similarly, CBS participants showed a slightly delayed BOLD signal in the motor hand area (optimal lag: 0 TR) compared to sighted controls (optimal lag: −0.47 TR, *P *<* *0.001, two-tailed permutation test) in the scans involving manual report of hallucinations. A significant interaction between groups (CBS, sighted controls) and regions of interest (early/intermediate visual cortex, lips region: *P *<* *0.001; early/intermediate visual cortex, hand region: *P *<* *0.001, two-tailed permutation tests) confirmed that the early build-up of activity in the CBS compared to the sighted control group was specific to the visual cortex.

Supplementary Figs 7 and 8 present the temporal dynamics in the brains of single CBS participants compared to the relevant sighted controls' data, based on events locked to the CBS or sighted controls’ report.

### Signals preceding hallucinations diminish across the visual hierarchy

**Figure 5:**
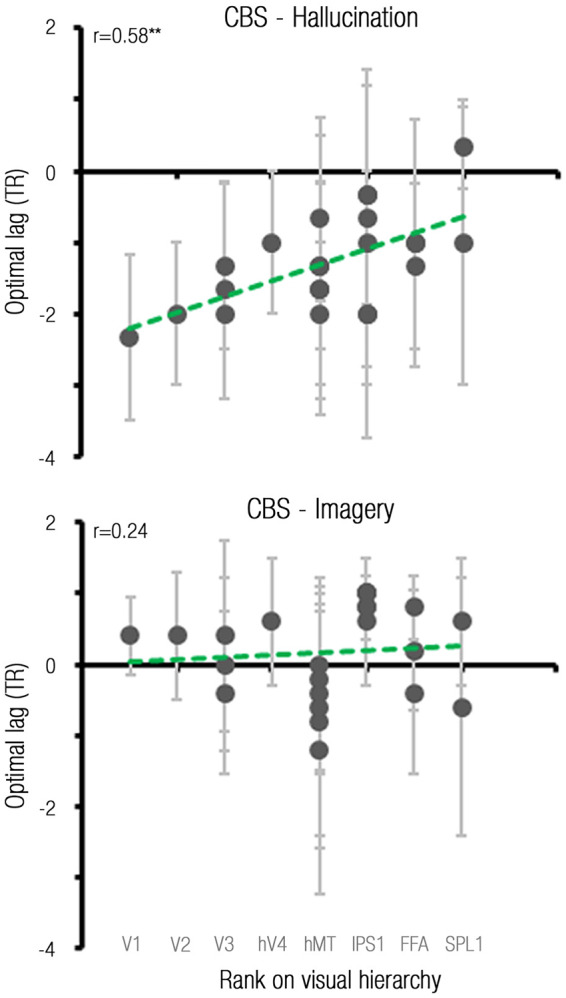
**Hallucination-related preceding signals decay along the visual hierarchy.** The *top* and *bottom* plots present the data of the CBS group in the hallucination and imagery conditions, respectively. *X*-axes represent the hierarchical rank of visual regions of interest (Supplementary Table 1), grey labels depict one representative region of interest from each ranking category. *Y*-axes represent the optimal lag of the HRF relative to stimulus onset, as fitted to signals from these regions of interest. Dots depict group means for regions of interest and error bars depict standard deviations. IPS = intraparietal sulcus; SPL = superior parietal lobe.

The above analyses of our data revealed that although hallucinations engage the entire visual system, the extent to which neural activity builds up prior to hallucination may vary across visual brain regions. We therefore aimed to examine whether there was a trend in the ‘propagation’ of this preceding signal across the visual hierarchy. Of importance, when measuring evoked visual responses, synaptic lags along the cortical hierarchy are too rapid to be captured using functional MRI (Dijkstra *et al.*, 2020). However, since spontaneous brain activity is composed of ultra-slow fluctuations (Nir *et al.*, 2008), its decay across the visual system could be captured despite the sluggishness of the BOLD signal. Indeed, as depicted in Fig. 5, a significant correlation was found in the CBS group during hallucination, such that lower-level visual areas demonstrated an earlier build-up of activity relative to hallucination report, compared to higher-order visual areas (*r *=* *0.58, *P = *0.004, two-tailed permutation test, see Supplementary Table 3 for correlations in single CBS participants).

To evaluate whether the ordered propagation of preceding signals across the visual system is distinctive of hallucinations in participants with CBS, we tested for a correlation between visual ranks and optimal lags in the CBS group during the imagery condition, and found no evidence for such a relation (*r *=* *0.24, *P = *0.13, two-tailed permutation test).

## Discussion

Here, we demonstrate that visual hallucinations, much like any other type of visual experience, engage the entire visual system. We additionally report that, unlike other types of visual experiences, visual hallucinations are related to a spontaneous build-up of neural activity, which is most pronounced in early visual cortex, and decays along the visual hierarchy. Taken together, our findings propose a network view of visual hallucinations in CBS, under which a slow build-up of spontaneous neural activity in early visual regions may bring about a perception of vision by triggering a cascade of activity throughout the visual system.

Our findings suggest a plausible mechanism underlying the emergence of visual hallucinations in CBS. Specifically, deafferentation of the visual system may lead to over-excitability in visual areas (Desai *et al.*, 1999; Burke, 2002; Reichert *et al.*, 2013; Painter *et al.*, 2018), and to enhanced susceptibility to noise fluctuations. Given the normally-developed hierarchical structure of the visual system in (previously sighted) individuals with CBS, when an activation threshold in early visual regions is crossed by a spontaneous fluctuation, a cascade of activations across the visual hierarchy may lead to the emergence of visual hallucinations (Moutard *et al.*, 2015). Further imaging studies are needed for clarifying the unique characteristics of spontaneous brain activity in individuals with CBS compared to non-hallucinating blind individuals.

Unlike our findings, studies of sighted patients with dementia-related visual hallucinations reported a reduction in visual activation (Meppelink *et al.*, 2009; Goetz *et al.*, 2014), which may indicate that hallucinations are a product of impaired bottom-up processing of external visual stimuli. However, we propose that hallucinations in CBS are related to intact bottom-up visual processing, but are a product of internal, rather than external, visual inputs (as individuals with CBS are visually deprived). Thus, our findings do not offer a unified account for visual hallucinations across many disorders, but instead illuminate the possible role of spontaneous brain activity in evoking visual hallucinations consequent to visual deprivation.

In this interpretation, we assume that the activity seen in the visual system of blind individuals is spontaneous (Echlin *et al.*, 1952; Loeser and Ward, 1967; Segal and Furshpan, 1990). Yet, it is also possible that the visual system of these individuals is activated by inputs from other brain areas processing non-visual information (cross-modal plasticity; Edelman, 1993; Amedi *et al.*, 2003; Collignon *et al.*, 2011; Renier *et al.*, 2014). However, the extent of cross-modal plasticity in the late blind is still debated (Collignon *et al.*, 2013; Voss, 2013). Additionally, hallucinations in our sample of CBS participants appeared in the absence of any external sensory trigger, suggesting that sensory inputs did not elicit the hallucinations.

It could also be argued that the slow build-up of activity observed in the hallucination condition may simply reflect a delay between hallucination onset and its report, due to the elusive nature of the hallucination. However, all CBS participants included in this condition indicated that they could report their hallucinations as promptly as they could report visual stimuli before their vision deteriorated. Moreover, if this hypothetical confound actually held, hallucinations should evoke an increase in BOLD signal, similarly to veridical vision (Fig. 1B). A consequence of this is that there should be rapid propagation of activation across the visual system (Dijkstra *et al.*, 2020) leading to an ‘artificial build-up’ of activity across the entire visual hierarchy. However, this possibility is contradicted by our findings of a decaying activity build-up across the visual hierarchy (Figs 4 and 5). It is therefore more likely that the anticipatory signals reflect accumulation of spontaneous activity prior to hallucination onset, rather than hallucination-evoked activity which precedes the onset of report.

While our findings indicate an endogenous trigger for visual hallucinations in CBS, a recent EEG study proposed that hyperexcitability in the early visual cortex of elderly, partially blind CBS individuals, is related to external visual stimulation (Painter *et al.*, 2018). Another case study of an elderly individual with CBS and mild sensorineural deafness ascribed hallucinations to external auditory stimulations of the early visual cortex (Vacchiano *et al.*, 2019). Given the difficulty in recruiting large samples of CBS participants (Teunisse *et al.*, 1996; Plummer *et al.*, 2007; Cox, 2014), and given the demographic and clinical differences in the recruited samples in the three studied, we cannot exclude the possibility of a heterogeneous mechanism for hallucinations, combining both internal and external stimulation of the hyperexcitable early visual cortex. However, in our sample of participants, the observation of a slow, anticipatory build-up of activity in the visual cortex prior to hallucination onset cannot be accounted for by external stimulation. Our study clearly demonstrates that hallucinations in CBS can be evoked in the absence of external visual information.

As such, our findings provide unique insight into the possibility that spontaneous brain activity can evoke conscious percepts by triggering existing neural cascades. This observation is compatible with previous hypotheses regarding the role of the slow spontaneous activity in initiating internally-generated behaviour (Schurger *et al.*, 2013; Moutard *et al.*, 2015), which could be directly tested here due to the unique characteristics of CBS. Taken together, these findings raise the intriguing possibility that spontaneous network dynamics may underlie many types of internally-generated behaviours. For example, it is tempting to conjecture that if accumulation of spontaneous activity in the visual system of blind individuals can ignite visual hallucinations, a similar mechanism may also be implicated in more typical forms of unprompted and deprivation-related percepts, such as in dreaming.

### Limitations

Since CBS is rare, our sample size was small and heterogeneous in both the phenomenology of hallucinations, and in neural responses to visual hallucinations. Some of the small reported effects may therefore relate to a reduction in statistical power. It is worth noting, however, that despite the heterogeneity, all three participants evinced the same activation profile with the slow build-up of activation in early visual cortex. Additionally, we acknowledge that it is impossible to simulate accurately the content/timing of hallucinations because (i) our only access to the visual experiences of the CBS participants was the participants’ subjective reports; (ii) the realistic 3D attributes of the hallucinations could not be simulated when presented on a computer screen; and (iii) our simulated stimuli were much smaller and more confined to the fovea compared to the real hallucinatory percepts, as we needed to provide enough movement space for stimuli on the display screen. Nevertheless, we made every effort to capture the gist and the reported temporal dynamics of the hallucinations.

## Supplementary Material

awaa384_Supplementary_Data

## Abbreviations

BOLD =: blood oxygenation level-dependent
CBS =: Charles Bonnet syndrome
FFA =: fusiform face area
HRF =: haemodynamic response function
TR =: repetition time
