# Supplementary material for: How do the blind ‘see’? The role of spontaneous brain activity in self-generated perception
Source: Brain. 2020 Dec 26;144(1):340–53. doi: 10.1093/brain/awaa384 (PMC7880672; doi:10.1093/brain/awaa384)
Supplement: awaa384_Supplementary_Data [file awaa384_supplementary_data.pdf]

## Supplementary Methods

### Hallucination phenomenology

CBS participant1 (CBS1) reported hallucinations consisting of still, coloured images of varying categories, including humans, animals, body-parts, objects, houses etc. The hallucinations contained both realistic and non-realistic (e.g. drawings) images, which appeared in varying locations of the visual field. The hallucinations were continuous (no periods in which no image was present).

CBS2 reported hallucinations consisting of one vivid but unfamiliar male face, which could blur or sharpen, and could rotate and move around the participant's visual field. This face appeared and disappeared spontaneously. Occasionally, a pink stain also appeared in the participant's visual field.

CBS3's hallucinations comprised of flashes of light, clouds, inkblots and various faces, which were described as either regular or "ghost-like". The faces were mostly in motion across the participants' visual field. Some occurrences of hallucinated objects (ball, flower) were also reported. When hallucinations appeared, they tended to be continuous and last for long periods of time (several hours to days). Sometimes the hallucination stream could be interleaved with periods of no hallucinatory content.

CBS4 reported the presence of very rapid flashes of light that spanned the entire visual field.

CBS5 described hallucinations made of rapidly changing patterns and colours, resembling a kaleidoscope. These varying patterns were present simultaneously in different locations of the visual fields.

### Experimental software

All lights in the scanning room were off during the experiment. In addition, in the CBS and Blind control scanning sessions, there was also no illumination from the display screen. Stimulus presentation and button press recordings were implemented in Matlab using the Psychophysics Toolbox extensions (Brainard, 1997) and also using the Presentation® software (Version 18.0, Neurobehavioral Systems, Inc., Berkeley, CA, www.neurobs.com). In-scanner voice recording of the CBS participants were carried out using Optoacoustics Microphone 2.4 (Optoacoustics Ltd.).

## MRI data acquisition

All scans were acquired with a 3T Trio MRI Siemens scanner equipped with a 12 channel head matrix receiving coil at the Weizmann Institute of Science, Rehovot, Israel. Functional images of blood oxygenation level dependent (BOLD) contrast were obtained using a T2* weighted gradient echo planar imaging (EPI) sequence (TR = 2000 ms, TE = 30 ms, flip angle = 75°, FOV 228 mm, voxel size 3 × 3 × 4 mm, 31 slices, tilted to the ACPC plane). Anatomical data were acquired using a T1-weighted magnetization prepared rapid acquisition gradient echo (MPRAGE) sequence (TR = 2300 ms, TE = 2.98 ms, TI = 900 ms, flip angle = 9°, voxel size 1 × 1 × 1 mm).

## Preprocessing of functional data

All fMRI data were processed using FSL 5.0.2.1 (www.fmrib.ox.ac.uk/fsl) and in-house Matlab code (Mathworks, Natick, MA, USA). Functional data were analyzed using FMRIB's expert analysis tool (FEAT, version 6). The following preprocessing steps were applied to each participant's data: motion correction using FMRIB's Linear Image Registration Tool (MCFLIRT; Jenkinson et al., 2002), brain extraction using BET (Smith, 2002), high pass temporal filtering of 100 s and spatial smoothing using a Gaussian kernel with a FWHM of 5 mm. Functional images were aligned to structural images initially using FMRIB's Linear Image Registration Tool (FLIRT; Jenkinson and Smith, 2001; Jenkinson et al., 2002) and then optimized using Boundary-Based Registration (BBR; Greve and Fischl, 2009). A stringent approach was taken for the handling of head movements that may have been induced by in-scanner speech. Specifically, the motion parameters of each participant were inspected to identify severe head movements. Scans with head motion exceeding 1.5 mm were removed from subsequent analyses. Small head movements that might impact on the BOLD signal were identified using a scrubbing procedure (Power et al., 2012). Scans in which more than 50% of volumes were found to contain motion were removed from subsequent analyses. Structural images were transformed into MNI space using FMRIB's Nonlinear Image Registration Tool (FNIRT). Tissue-type segmentation was carried out using FAST (Zhang et al., 2001) to create white matter/CSF nuisance masks. To avoid the inclusion of grey matter voxels in these nuisance masks, these masks included only voxels identified as white matter/CSF with probability of 1, restricted by an anatomically-based bounding box (-44<x<42, -84<y<42, -4<z<34 for white matter; -42<x<38, -64<y<38, -22<z<28 for CSF), and eroded by a sphere of radius 5 and 2 mm for white-matter and CSF, respectively (Hahamy *et al.*, 2017). White-matter and CSF time-courses were extracted for each

functional scan, and their contribution to the BOLD signal, as well as the contribution of motion parameters, was later removed (see Statistical Analyses).

## GLM designs

To create task-based statistical parametric maps, we applied a voxel-based general linear model (GLM) as implemented in FEAT, using a double-gamma hemodynamic response function convolved with the experimental model, as well as the resulting regressors' temporal derivatives. The six motion parameters and their derivatives, scrubbed volumes (Power et al., 2012), and ventricle and white-matter time-courses for each participant (Fox et al., 2009) were used as nuisance regressors. In addition, in the simulated-hallucinations data, the first/last 5 TRs of each scan were included in the GLM model as a nuisance variable, to remove the contribution of arousal-related effects.

In cases where a single participant had multiple scans of the same experiment (multiple hallucination scans of each CBS participant, verbal/button-press report scans of the same hallucination-simulation stream in Sighted participants, two imagery scans for CBS and Blind controls), the whole-brain parametric maps resulting from each scan were submitted to an FFX analysis, as implemented by FEAT. Z (Gaussianized T/F) statistical images were then thresholded using clusters determined by $Z>2.3$ ($p<0.05$), and a family-wise-error corrected cluster significance threshold of $p<0.05$ was applied to the suprathreshold clusters.

Within-group analysis of statistical maps was carried out using FMRIB's Local Analysis of Mixed Effects (FLAME1 for controls and FLAME1+2, suitable for small sample sizes, for the CBS group). Z (Gaussianized T/F) statistic images were thresholded using clusters determined by $Z>2.3$ ($p<0.05$), and a family-wise-error corrected cluster significance threshold of $p<0.05$ was applied to the suprathreshold clusters.

### GLM design of hallucination and simulated hallucinations

The unique and complex hallucinatory contents participants reported during scanning required a different modelling approach for each CBS participant, as detailed below.

CBS1 hallucinated rapidly changing images, without any hallucination-free periods. Due to the absence of a baseline, this hallucinatory stream was modelled using transients – regressors of 1 second that marked the reported appearance of each image. This same approach was used for both the verbal and button-press reports of this participant.

CBS2 hallucinated a single face, appearing for relatively long periods, in which the face blurred and sharpened. This hallucinatory stream was modelled using a parametric block design, assigning more predicted activation to periods in which the face appeared clearly compared to periods in which it was blurred. Since the non-verbal report of this participant was comprised of only 2 types of button-presses (one for appearances and the other for disappearances of the hallucinated face), a non-parametric block-design was used to model the manually reported hallucination-stream.

During the verbal-report scan, CBS3 hallucinated faces, objects and patterns, each appearing for relatively long periods without any hallucination-free intervals. Due to the absence of a baseline, this hallucinatory stream was modelled using transients – regressors of 1 second that marked the reported appearance of each hallucination. The non-verbal report of this participant comprised of reports of faces using 2 types of button-presses (one for appearances and the other for disappearances). Since a post-hoc inquiry revealed that these faces were sometimes followed/preceded by other hallucinatory contents and, at other times, by no hallucinations at all (hence the reported disappearance of a face was not indicative of an appearance of a novel perceptual content), only reports of face appearances were modelled as transients.

The exact same design used for each CBS participant was also used to model the Sighted controls' data in the relevant simulated hallucination conditions. However, note that the hallucination-simulation was constructed such that it presented the hallucinatory content 1 second earlier than reported by the CBS participants. The use of the same design locked to the onset of hallucinations as reported by the CBS participants was thus assumed to compensate for inaccuracies in the reported timing of hallucinations due to the reaction times of CBS participants (Ben-Yakov and Henson, 2018). Note that this temporal difference in designs between groups is in the opposite direction to our hypothesis: as illustrated in Figure 1, we expected to find that the Sighted controls' signals would rise after those of the CBS participants, although the events in the control group had preceded those of the CBS group. Thus, results that are compatible with our hypothesis cannot be explained by the group difference in designs.

Nevertheless, in order to verify that no reaction-time related biases were introduced into our analysis, we also measured brain activation locked to the individual reports of Sighted controls, using only the scans in which Sighted controls reported the content of the simulated

hallucinations using registered button-presses. As in the previous analysis, these button-presses were modelled as transients in the simulated hallucinations of CBS1 and CBS3, and as a block design for the simulated hallucinations of CBS2.

In all analyses, we used two contrasts of interest: vision>baseline (marked in warm colors in our figures) and vision <baseline (marked in cold colors in our figures).

As noted above, it is impossible to accurately simulate the internal visual experiences of the CBS participants. Due to the inherent differences between the experimental conditions of the two groups, we refrained from directly comparing activation strengths between groups (as any effects could be equally attributed to differences between hallucinations and veridical vision, or to differences in the experimental conditions). Instead, within-group whole-brain maps were used for qualitative inference, and a between-group comparison of similarity in the spatial patterns of visual activation was made (see section “Quantifying the similarity between visual activations”).

### GLM design of visual imagery

This experiment was modelled as a block design with four conditions: faces, houses, objects and patterns. The two contrasts of interest were the overall positive activation evoked by imagery across all categories (imagery>baseline) and the overall negative activation evoked by imagery across all categories (imagery<baseline).

### GLM design of the verbal-manual control condition

This experiment was aimed to isolate the neural correlates of verbal and manual reports, in order to ensure that report-related brain activations do not underly any effects observed during visual hallucinations in CBS participants. This event-related design had two conditions, which were based on the actual responses made by participants: events where a button press was registered were included in the manual condition, and all other events were included in the verbal condition (participants were monitored during the scan to make sure there was indeed a response for each stimulus). The two contrasts of interest were the overall positive activation evoked by either type of response (verbal+manual reports>baseline) and the overall negative activation evoked by either type of response (verbal+manual reports<baseline).

## Quantifying the similarity between visual activations – bootstrap analysis

To ensure that the measured correlations between the CBS hallucination-related activity and the controls’ activity during the other experimental conditions were not merely driven by

noise, we employed a bootstrap analysis to quantify the distribution of noise correlations in our data. As previously described, each sighted control watched three simulated hallucinations, and each simulated hallucination had an experimental protocol locked to the onset of events. In the current analysis, the null hypothesis was that activation similarities between the sighted and CBS participants are not driven by the experimental protocols, but rather by noise. Under this hypothesis, the experimental protocols were shuffled between the imaging datasets acquired during the viewings of the three simulated hallucinations, such that each imaging dataset was entered into a separate GLM analysis with each of the three CBS experimental protocols (along with the same nuisance regressors described in the main analysis) as a design matrix. The same GLM analysis described in the main methods was repeated here, resulting in three beta maps (corresponding to the protocol of CBS1/2/3) for each single-subject dataset. Next, for each participant, three randomly selected maps (one from each dataset) were entered into an FFX analysis. This yielded a single random simulated-hallucination map for each participant, as in the main analysis. The posterior brain beta values of this map were correlated with the real posterior brain beta values from the CBS hallucination group map, and the resulting correlation coefficients were averaged across the control participants. This procedure was repeated 10,000 times to create the null distribution, and the original (unshuffled) averaged correlations across participants was used to derive the test's p-value.

For each of the imagery and verbal-manual control conditions, two random experimental protocols were created, maintaining the temporal structure and number of events in each condition. These two random design matrices, along with the original one, were used as regressors in separate GLM analyses for the dataset of each sighted/blind control participant and experimental condition, and the same procedure described for the simulated-hallucination condition was repeated, to evaluate whether the similarities between these conditions and the hallucination conditions were significantly greater than those induced by noise.

## Regions Of Interest (ROIs) definition

Our experimental hypothesis, that a build-up of activity across the visual hierarchy precedes the emergence of hallucinations, was tested using two planned comparisons across groups: to sample the bottom of the visual hierarchy, we chose an inclusive early/intermediate visual area. To sample the top of the hierarchy, we chose the Fusiform Face Area (FFA), both because all reporting CBS participants hallucinated faces (see Table 1) and because the fusiform gyrus has been previously associated with CBS hallucinations (Ffytche *et al.*, 1998).

The definition of the early/intermediate visual ROI was based on the aggregation of areas V1-V4 from a probabilistic atlas (Wang *et al.*, 2015). Specifically, for each of the V1-V4 regions separately, the 200 voxels of highest atlas probability scores were selected. The lateral and medial aspects of V1, V2 and V3 were joined together to form a unified ROI for each of these regions (no lateral/medial differentiation exists for area V4 in the atlas). In addition, homologous ROIs across the two hemispheres were joined to create a bilateral definition of ROIs. Then, all V1-V4 regions were merged together to form the final early/intermediate visual ROI.

The probabilistic atlas used lacked a definition of the FFA. This ROI was therefore defined bilaterally based on an independent dataset acquired from sighted participants undergoing a visual localizer task (Wilf *et al.*, 2017). This ROI was defined by the contrast of faces>patterns at a statistical threshold of Z=3, and consisted of 521 voxels.

A bilateral lips ROI and unilateral hand ROIs (to match the handedness of each participant) were created to serve as non-visual control ROIs, and were defined based on an additional independent dataset of typical control participants undergoing a body localizer task (Hahamy *et al.*, 2015). Left/right hand ROIs were defined from the contrast of left/right hand movements>rest at a statistical threshold of Z=6.5, and consisted of 455/429 voxels, respectively. The bilateral lips ROI was defined by the contrast of lip movements>rest at a statistical threshold of Z=6.5, and consisted of 562 voxels.

Finally, to evaluate the possible propagation of activity across the visual hierarchy, we used all regions of the probabilistic atlas. ROIs consisted of the 200 voxels of highest atlas probability scores in each functional region. The lateral and medial aspects of V1, V2 and V3 were joined together to form a unified ROI for each of these regions. In addition, homologous ROIs across the two hemispheres were joined to create a bilateral definition of ROIs.

## Testing for activation in the early/intermediate visual ROI

To test whether the early/intermediate visual ROI showed a significant signal increase in the CBS group, the following permutation test was employed. For each CBS participant, the area under the curve of the event-related averaging signal of this ROI was calculated, bounded by the optimal point of signal increase (as determined by the optimal lag analysis, see Supplementary Table 2) and the following 4 TRs (the period of positive signal change, determined by the imagery condition). The test statistic was set as the averaged values across the 3 CBS participant. Under the null hypothesis that hallucinations do not result in BOLD

signal increase, the timing (protocol) of reported hallucinations could be shuffled within each individual CBS participant and reporting scan. To construct this null distribution experimentally, the same event-related analysis described in the main Methods was repeated for each CBS participant using 10,000 shuffled protocols, and the area under the curve was calculated for each resulting random event-related signal. This procedure yielded 10,000 triplets of activity levels for the 3 CBS participants under random protocols, which were averaged across participants to form the null distribution. The test's statistic was then compared to this null distribution to obtain a p-value.

To further substantiate the observation that the between-group difference in optimal lag exists in highly activated portions of the early visual cortex, an additional approach was unitized. The early visual ROI (defined based on a probabilistic atlas acquired from sighted participants) was intersected with the positive and significant activations of the CBS hallucination map (Figure 2). The resulting ROI consisted of 621 voxels. Having defined this ROI based on voxels of significant BOLD signal change, we could next test whether this ROI also showed a significant difference in optimal lag between the CBS and Sighted control groups (as described in the main Methods), to ensure that a build-up of activity is observed in voxels most strongly activated by hallucinations in the CBS groups.

## Evaluation of temporal dynamics – permutation tests

The "optimal lags" of the HRF in each ROI were compared between the CBS group and each of the Sighted/Blind control groups separately, using a permutation test. For the imagery-related data, the test statistic was set as the difference between the group's mean optimal lags in a certain ROI. Under the null hypothesis of no difference in optimal lags between groups, participants' labels (CBS or Sighted/Blind control) were permuted. Thus, two random experimental groups were created, and the difference between the groups' mean optimal lags in a given ROI was calculated. This procedure was repeated for all possible combinations of participants between the two groups, constructing the full null distribution of group differences. For each ROI, the position of the true (unshuffled) group-difference relative to the null distribution was used to obtain a two-tailed p-value.

In order to compare the optimal lags between the hallucination and simulated hallucination conditions in each ROI, a different approach was used. This approach took into consideration the unique stream of events that is shared between the hallucinations of each CBS participant and the Sighted controls who experienced his/her simulated hallucinations. The null

hypothesis here was of no difference in optimal lags between each CBS participant and the Sighted controls who experienced his/her simulated hallucinations, and therefore participants' labels (CBS or Sighted control) could be permuted. To this end, the data of each CBS participant and Sighted controls watching his/her simulated hallucinations were considered as a homogenous group. For each participant in this group, the difference between the participant's optimal lag and the averaged optimal lag across all other participants was calculated. These difference-scores were calculated separately for each of the three groups corresponding to the hallucinatory stream of each of the three CBS participants. The chosen test statistic was the average of difference-scores across the three (real) CBS participants. To create the null distribution, three random participants, one from each group (including both the CBS and Sighted control participants of each group) were chosen, and their average difference-scores were calculated. This procedure was repeated for all possible combinations of participants across the three groups, creating random averaged difference-scores that constructed the full null distribution. For each ROI, the position of the true averaged CBS difference-score relative to the null distribution was used to obtain a one-tailed p-value, to test whether the CBS hallucinatory-related BOLD signal indeed precedes the onset of hallucinations, unlike the BOLD signal of Sighted controls.

Interactions between the factors group (CBS, Sighted controls) and either condition (hallucinations/simulated hallucinations, imagery) or ROI (visual, sensorimotor) were evaluated using permutation tests. Under the null hypothesis of no 2-way interactions between groups and conditions/ROIs, both participants' group labels and within-participant condition/ROI labels were permuted. Within-participant differences were calculated and averaged across participants of the same experimental group, and mean group differences were derived. The position of the true group-difference relative to the null distribution (resulting from 10,000 such iterations) in each experimental condition was used to derive a two-tailed p-value.

# Supplementary Tables

**Supplementary Table 1. Related to Figure 5. List of visual regions, and their assigned rank across the visual system.** Regions were taken from a probabilistic atlas (Wang et al., 2015), with the exception of the FFA, which was added to this list and independently defined based on data published in Wilf et al., 2017.

| Visual ROI | Rank |
|---|---|
| V1 | 1 |
| V2 | 2 |
| V3 | 3 |
| V3b | 3 |
| V3a | 3 |
| hV4 | 4 |
| VO1 | 5 |
| VO2 | 5 |
| MST | 5 |
| hMT | 5 |
| LO2 | 5 |
| LO1 | 5 |
| IPS0 | 6 |
| IPS1 | 6 |
| IPS2 | 6 |
| IPS3 | 6 |
| IPS4 | 6 |
| IPS5 | 6 |
| PHC1 | 7 |
| PHC2 | 7 |
| FFA | 7 |
| SPL1 | 8 |
| FEF | 8 |

**Supplementary Table 2. Related to Figure 4. Optimal HRF lags in single CBS participants.** Rows represent experimental condition (Hallucination\imagery) and ROIs activated by these conditions. Columns present the three reporting CBS participants. Lags are given in TRs.

| condition | ROI | CBS1 | CBS2 | CBS3 |
|---|---|---|---|---|
| **Hallucination report** | Early/intermediate visual | -3 | -1 | -3 |
| | FFA | 0 | 0 | -3 |
| | Lips | 0 | 0 | 1 |
| | Hand | 0 | 0 | 0 |
| **Imagery** | Early/intermediate visual | 1 | 1 | 1 |
| | FFA | 1 | 1 | 1 |

**Supplementary Table 3. Related to Figure 5. Correlations (Spearman's rho and two-tailed p-values) between the ranks of visual ROIs and optimal HRF lags across ROIs in single CBS participants.**

| | **Spearman's rho** | **p-value (permutation test)** |
|---|---|---|
| **CBS participant 1** | 0.35 | 0.09 |
| **CBS participant 2** | 0.39 | 0.07 |
| **CBS participant 3** | 0.2 | 0.19 |

## Supplementary Figures

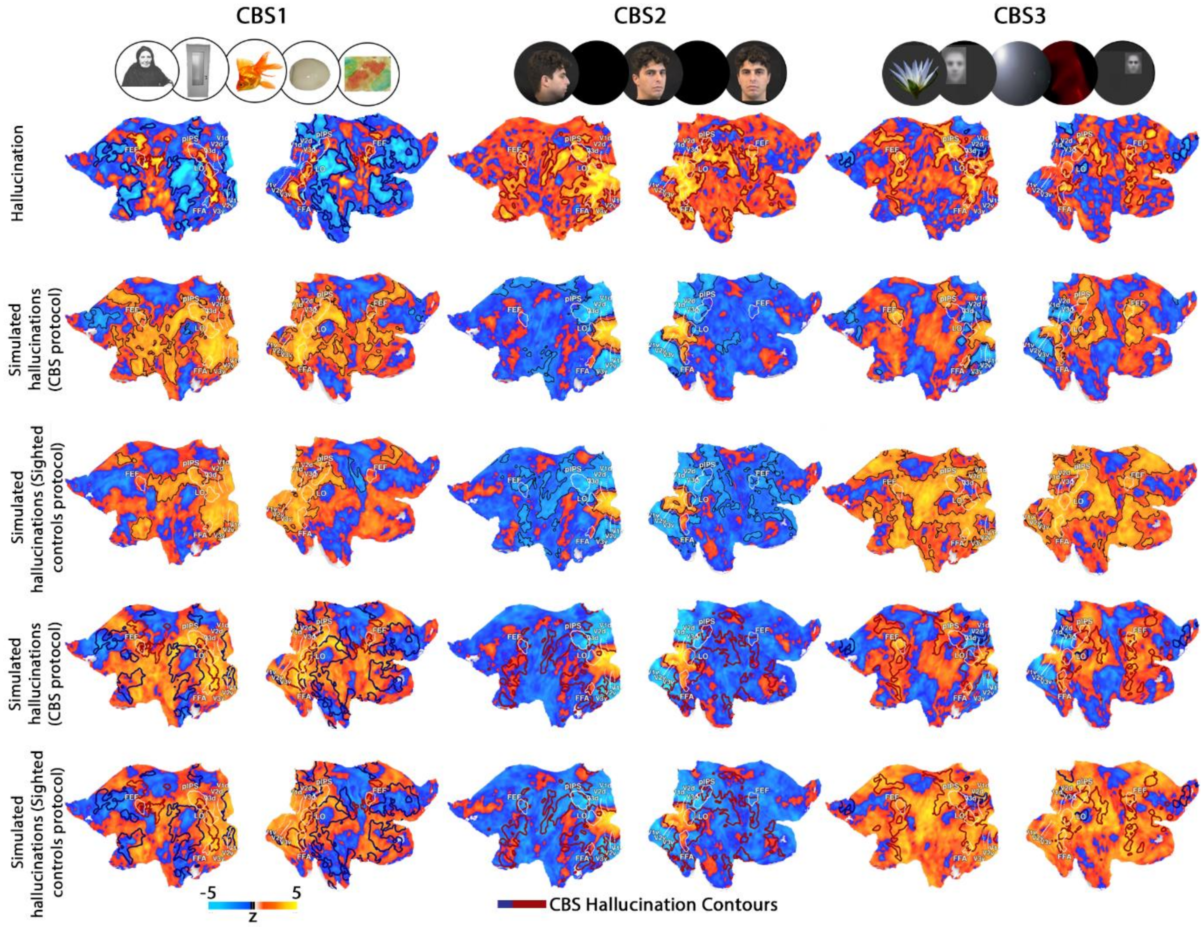


**Supplementary Figure 1. Related to Figure 2. Hallucination compared to simulation of visual hallucinations for single CBS participants.** Each column presents maps of one CBS participant and of the Sighted control participants who viewed an illustration of the hallucination stream of that CBS participant. The top of each column depicts examples of the hallucinatory streams of the CBS participants. First row: Unthresholded maps of single CBS participants in the hallucination condition (hallucination vs. baseline). Wide red/blue contours depict significant areas of activation/deactivation, respectively, corrected for multiple comparisons. Second row: Unthresholded group maps of the Sighted control group in the simulated-hallucination condition that matches the hallucinatory content of each CBS participant (visual stimuli vs. baseline). These maps are based on a GLM locked to one second prior to the onset of hallucinations, as reported by the CBS participants. Black contours depict significant areas of activation or deactivation, corrected for multiple comparisons. Third row: Unthresholded group maps of the Sighted control group in the simulated-hallucination condition that matches the hallucinatory content of each CBS participant. These maps are based on a GLM locked to the reported appearance of stimuli made by the Sighted controls (button-press reports only). Black contours depict significant areas of activation or deactivation, corrected for multiple comparisons.

Rows 4-5: the same maps as in rows 2-3, superimposed with the significant areas of activation/deactivation during hallucinations in the matching single CBS participants (wide contours, as in row 1). White contours depict visual landmarks, based on a probabilistic atlas. FFA, Fusiform Face area; LO, Lateral Occipital complex; pIPS, posterior Intraparietal Sulcus, FEF, Frontal Eye Field. See also supplementary Figure 2.

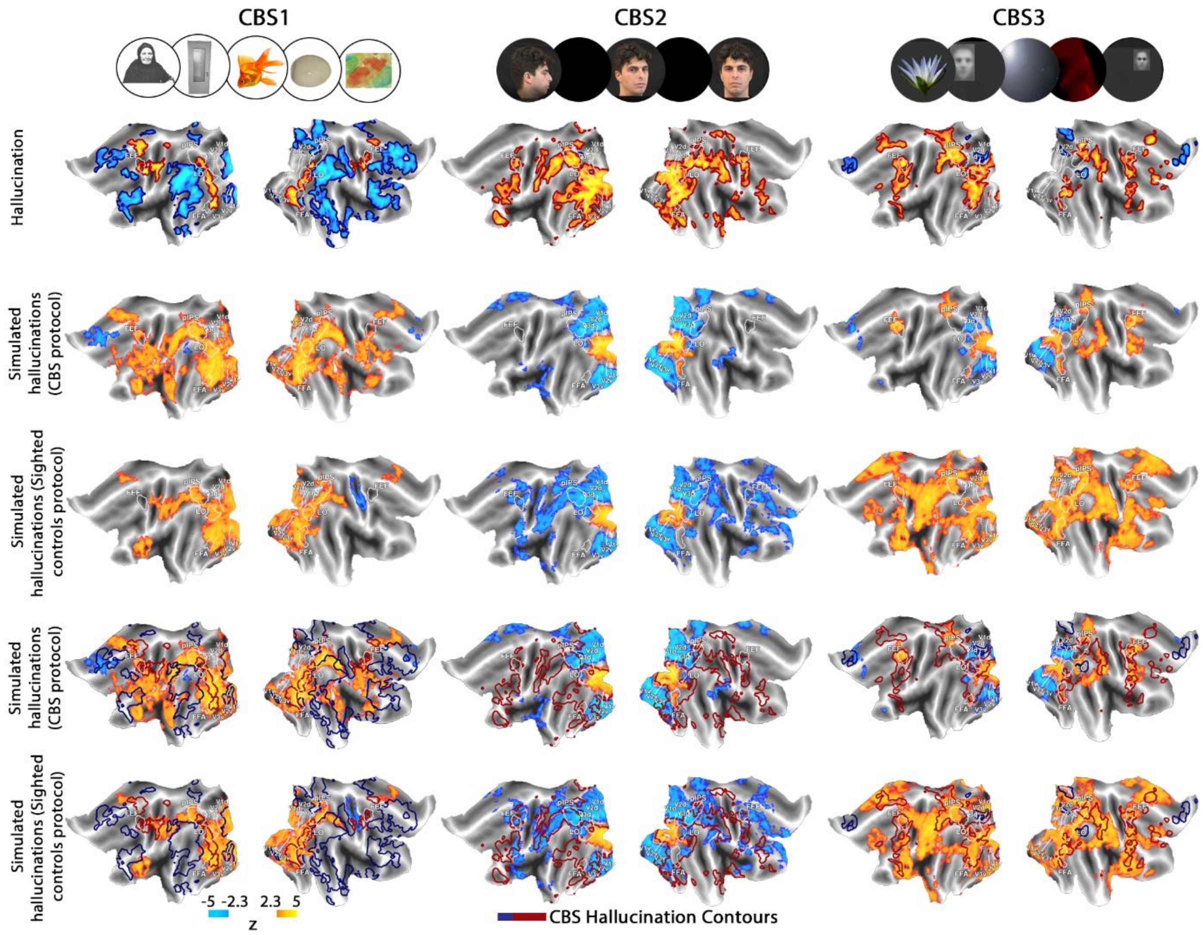


**Supplementary Figure 2. Related to Figure 2.** Same as Supplementary Figure 1, but presenting statistically thresholded maps, corrected for multiple comparisons.

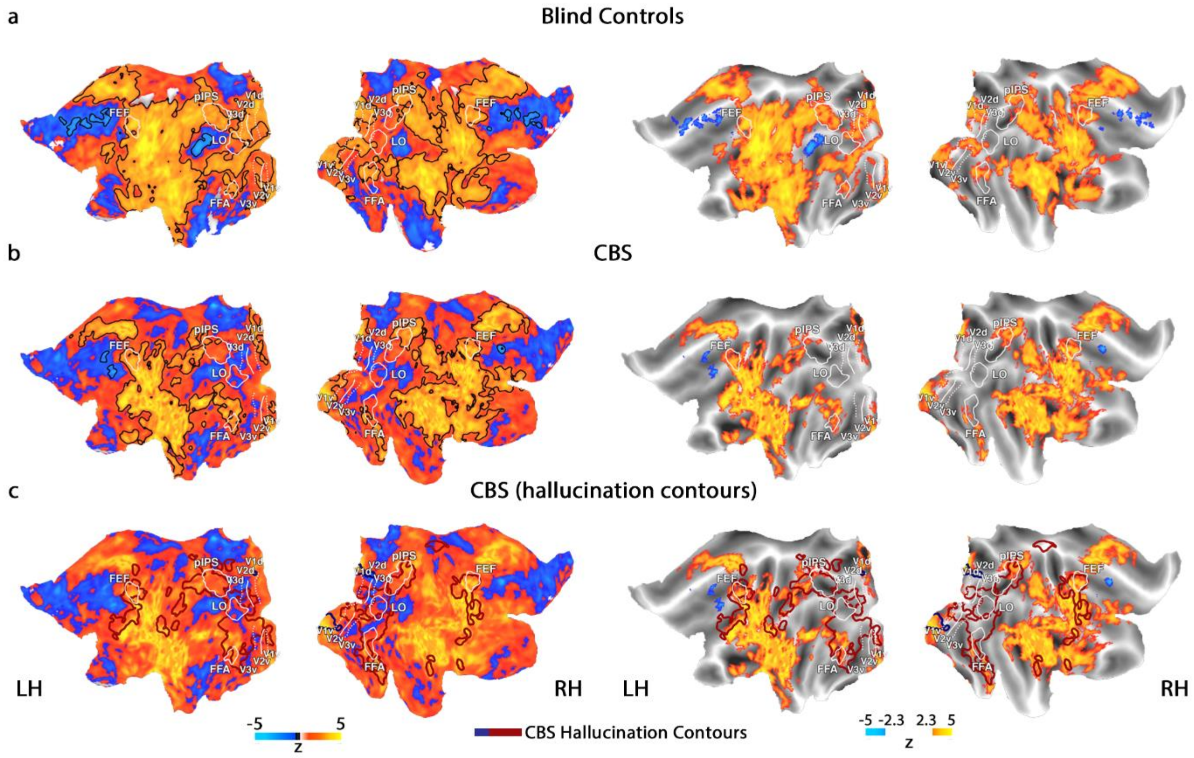


**Supplementary Figure 3. Related to Figure 2. Verbal-Manual control condition vs. Hallucination.** Left column presents unthresholded maps, right column presents the same maps with a statistical threshold, corrected for multiple comparisons. (a) Blind control group map during the control condition (presses+speech vs. baseline). Black contours depict significant areas of activation or deactivation, corrected for multiple comparisons. (b) CBS group map during the control condition (presses+speech vs. baseline). Black contours depict significant areas of activation or deactivation, corrected for multiple comparisons. (c) CBS group map during the control condition (as in (b)), superimposed with significant areas activated/deactivated during hallucinations (as presented in Figure 2, wide contours). Annotations are as in supplementary Figure 1. See also Supplementary Figures 4-5.

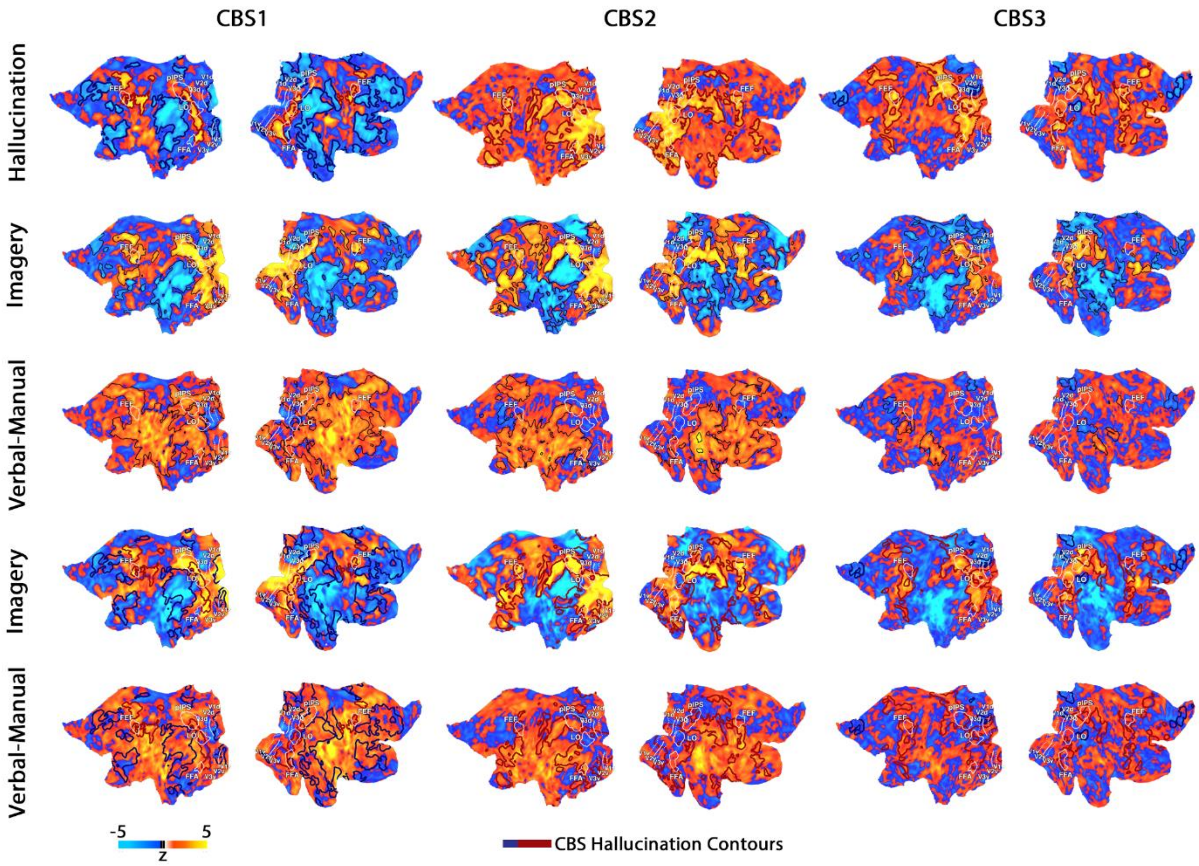


**Supplementary Figure 4. Related to Figure 3. Hallucination compared to visual imagery and to a verbal-manual control condition in single CBS participants.** Each column presents maps of one CBS participant. First row: Unthresholded maps of the hallucination condition (hallucination vs. baseline). Wide red/blue contours depict significant areas of activation/deactivation, respectively, corrected for multiple comparisons. Second row: Unthresholded maps of the visual imagery condition (imagery vs. baseline). Black contours depict significant areas of activation or deactivation, corrected for multiple comparisons. Third row: Unthresholded maps of the verbal-manual control condition (pressed+speech vs. baseline). Black contours depict significant areas of activation or deactivation, corrected for multiple comparisons. Rows 4-5: the same maps as in rows 2-3, superimposed with significant areas activated/deactivated during hallucinations in each CBS participant (wide contours, as in row 1). Note the similarity in the spatial overlap between hallucination and imagery activations in the posterior part of the brain across participants. Annotations are as in supplementary Figure 1. See also supplementary Figure 5.

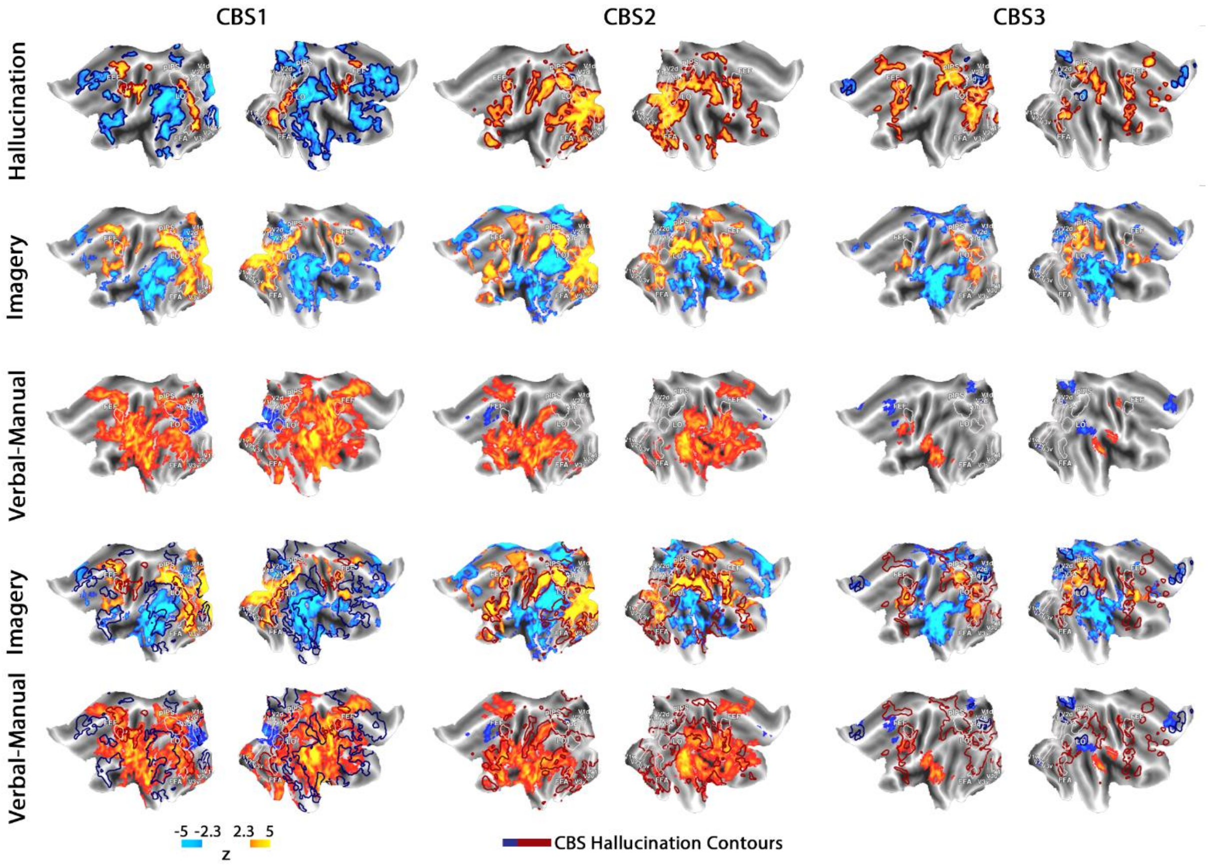


**Supplementary Figure 5. Related to Figure 3.** Same as supplementary Figure 4, but presenting statistically-thresholded maps, corrected for multiple comparisons.

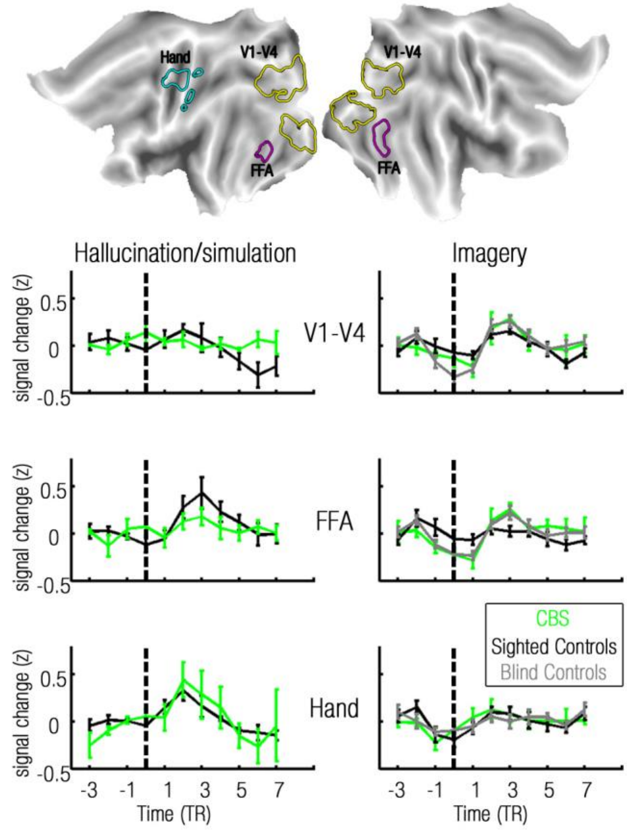


**Supplementary Figure 6. Related to Figure 4. Hallucination, vision and visual imagery have different temporal dynamics, results based on signals that are locked to the report of Sighted controls.** This figure presents the same results as in Figure 4, but the analysis of veridical vision is based on signals locked to the report (button-presses only) of Sighted controls, rather than on actual stimulus appearance (as reported by CBS participants). (a) Regions of interest (ROIs) – V1-V4 (yellow contours), FFA (purple contours), Hand area (light blue contours). Rows depict event-related averaging of group activations within the ROIs. Columns correspond with experimental conditions (Hallucination in CBS participants and Simulation of hallucination in Sighted Controls on the left column, Visual imagery in all groups on the right column). Groups are represented by green/black/grey lines for CBS/Sighted controls/Blind controls, respectively. Dashed lines depict reported hallucination onset (CBS) or reported stimulus appearance (Sighted controls) in the simulated hallucinations condition, and the onset of the auditory imagery instruction in the Imagery condition.

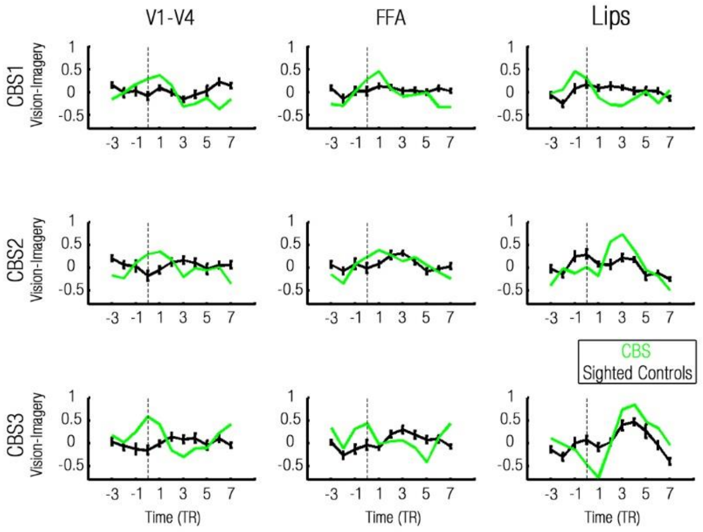


**Supplementary Figure 7. Related to Figure 4. Event-related averaging in single CBS participants.** The difference between vision-evoked and imagery-evoked signals is presented in each ROI (columns) and for each CBS participant (green) compared to the Sighted control group (black). ROIs are presented in Figure 4. Dashed lines depict reported hallucination onset (CBS) or the onset of stimuli in the simulated-hallucination condition (Sighted controls), and the onset of the auditory imagery instruction in the Imagery condition.

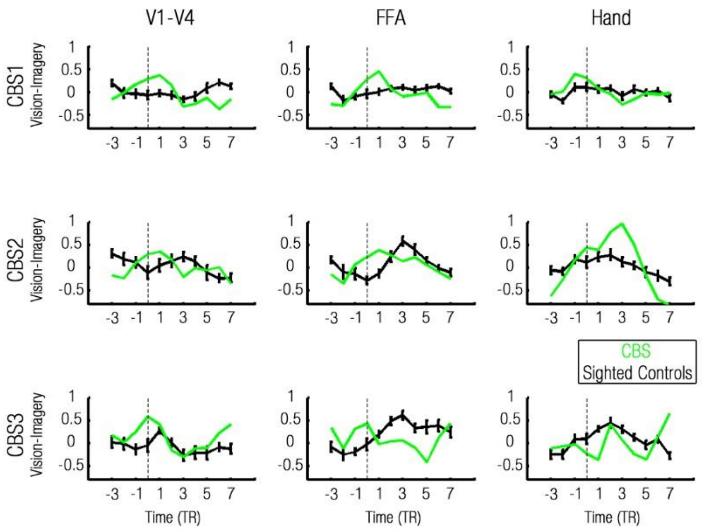


**Supplementary Figure 8. Related to Figure 4. Event-related averaging in single CBS participants, results based on signals that are locked to the reports of Sighted controls.** This figure presents the same results as in Supplementary Figure 7, but the analysis of veridical vision is based on signals locked to the report (button-presses only) of Sighted controls, rather than on actual stimulus appearance (as reported by CBS participants). The difference between vision-evoked and imagery-evoked signals is presented in each ROI (columns) and for each CBS participant (green) compared to the Sighted control group (black). ROIs are presented in Supplementary Figure 6. Note that here we used a hand ROI rather than the lip ROI, as in Supplementary Figure 6. Dashed lines depict reported hallucination onset (CBS) or reported stimulus onset (Sighted controls) in the simulated-hallucination condition, and the onset of the auditory imagery instruction in the Imagery condition.

## Supplementary References


Ben-Yakov A, Henson RN. The Hippocampal Film Editor: Sensitivity and Specificity to Event Boundaries in Continuous Experience. J Neurosci 2018; 38(47): 10057-68.

Brainard DH. The Psychophysics Toolbox. Spat Vis 1997; 10(4): 433-6.

Ffytche DH, Howard RJ, Brammer MJ, David A, Woodruff P, Williams S. The anatomy of conscious vision: an fMRI study of visual hallucinations. Nat Neurosci 1998; 1(8): 738-42.

Fox MD, Zhang D, Snyder AZ, Raichle ME. The global signal and observed anticorrelated resting state brain networks. J Neurophysiol 2009; 101(6): 3270-83.

Greve DN, Fischl B. Accurate and robust brain image alignment using boundary-based registration. Neuroimage 2009; 48(1): 63-72.

Hahamy A, Macdonald SN, van den Heiligenberg F, Kieliba P, Emir U, Malach R, *et al.* Representation of Multiple Body Parts in the Missing-Hand Territory of Congenital One-Handers. Curr Biol 2017; 27(9): 1350-5.

Hahamy A, Sotiropoulos SN, Henderson Slater D, Malach R, Johansen-Berg H, Makin TR. Normalisation of brain connectivity through compensatory behaviour, despite congenital hand absence. eLife 2015; 4.

Jenkinson M, Bannister P, Brady M, Smith S. Improved optimization for the robust and accurate linear registration and motion correction of brain images. Neuroimage 2002; 17(2): 825-41.

Jenkinson M, Smith S. A global optimisation method for robust affine registration of brain images. Med Image Anal 2001; 5(2): 143-56.

Power JD, Barnes KA, Snyder AZ, Schlaggar BL, Petersen SE. Spurious but systematic correlations in functional connectivity MRI networks arise from subject motion. NeuroImage 2012; 59(3): 2142-54.

Smith SM. Fast robust automated brain extraction. Hum Brain Mapp 2002; 17(3): 143-55.

Wang L, Mruczek RE, Arcaro MJ, Kastner S. Probabilistic Maps of Visual Topography in Human Cortex. Cereb Cortex 2015; 25(10): 3911-31.

Wilf M, Strappini F, Golan T, Hahamy A, Harel M, Malach R. Spontaneously Emerging Patterns in Human Visual Cortex Reflect Responses to Naturalistic Sensory Stimuli. Cereb Cortex 2017; 27(1): 750-63.

Zhang Y, Brady M, Smith S. Segmentation of brain MR images through a hidden Markov random field model and the expectation-maximization algorithm. IEEE Trans Med Imaging 2001; 20(1): 45-57.
